# The use of risk sharing tools for post adoption surveillance of a non pharmacological technology in routine practice: results after one year

**DOI:** 10.1186/1472-6963-13-181

**Published:** 2013-05-20

**Authors:** Carlos Campillo-Artero, Francisco M Kovacs

**Affiliations:** 1 Balearic Health Service, Palma de Mallorca, Spain; 2 Spanish Back Pain Research Network, Madrid, Spain; 3 Scientific Department, Fundación Kovacs, Palma de Mallorca, Spain

## Abstract

**Background:**

To report results obtained by combining risk sharing tools with post-adoption surveillance mechanisms in order to control quality of care and implement a value-based reimbursement scheme for Neuro-reflexotherapy (NRT), a non-pharmacological treatment proven effective for neck pain (NP), thoracic pain (TP) and low back pain (LBP).

**Methods:**

Pre-post prospective cohort study in routine clinical practice, carried out in primary care centers in the Spanish National Health Service in the Balearic Islands (Ib-Salut). Eight-hundred and seventy-one subacute and chronic NP, TP and LBP patients treated in Ib-Salut, who underwent NRT during 2011. A shared risk contract (SRC) was developed, where payments for NRT were linked to results on patients’ clinical evolution, reduction in medication and proportion of patients undergoing spinal surgery. Main outcome measures were local pain (NP, TP or LBP), referred pain, LBP-related disability and NP-related disability, measured using previously validated instruments at referral and 3 months later, use of medication assessed at referral and discharge, and rates of spinal surgery prescription after undergoing NRT.

**Results:**

Median improvements at discharge corresponded to 57.1% of baseline value for local pain, 75.0% for referred pain, 53.8% for LBP-related disability and 45.0% for NP-related disability. Patients taking medication at discharge represented 29.0% of those taking it at referral. The proportion of patients in whom spinal surgery was prescribed after undergoing NRT was 0%. These results were consistent with those from previous randomised controlled trials (RCTs) and studies in routine practice, and complied with the standards set in the SRC.

**Conclusions:**

It is feasible and effective to enhance post adoption surveillance methods with risk sharing tools to improve quality control and support value-based reimbursement decisions for NRT. The feasibility of generalising this approach to other settings and to other non-pharmacological treatments should be explored.

## Background

Clinical use of health technologies depends on regulatory frameworks which vary across countries. Nevertheless, the inappropriate use of both pharmacologic and non-pharmacologic procedures is widespread all over the world, leading to patients being unnecessarily exposed to potentially harmful procedures and risks, and to superfluous health costs [1-12].

The objective of shared risk contracts (SRCs) is that payers and providers share the risks deriving from the uncertainties associated with using a given health technology in routine practice. To this end, SRCs link payments to providers to whether results in practice reach pre-established goals. Providers may include health care providers or, more commonly, providers of products (e.g., the industry). The pre-established goals can be defined by levels of usage of a given technology, appropriateness of use, and/or clinical outcomes [13-18]. For instance, following a SRC payments for a new drug can be partially or totally reduced if clinical outcomes in routine practice do not reach the cut-off point previously established in the SRC.

Typically, SRCs have been developed as a pragmatic way of ensuring coverage for new drugs, usually highly expensive ones, designed to treat serious conditions, when: a) existing treatments have not shown to be effective, b) the evidence on the effectiveness and cost/effectiveness of the new drugs is either lacking or of low quality, and c) conducting high quality randomised controlled trials (RCTs) within a reasonable timeframe is unfeasible. Examples include conditions with a relatively low incidence or for which long follow-up periods are necessary for results to be clinically meaningful [19-21].

In these cases, SRCs allow the health system to provide treatments which are potentially effective or more cost-effective than existing alternatives, while: a) gathering data to assess whether the corresponding investment actually leads to expected outcomes (i.e., “coverage with evidence development” schemes), b) providing information for value-based pricing, c) limiting potential wastage of payers’ resources, by sharing costs with providers in the event that the technology does not reach expected outcomes, and d) strengthening post adoption surveillance.

When pharmacological or non-pharmacological technologies are implemented in routine practice, application conditions often vary from those used in RCTs. These differences may include training standards, indication criteria (such as “off label” use and other forms of treatment extension effects), and variations in compliance. As a result, outcomes (including adverse events) in clinical practice can deviate from those in previous RCTs.

Non-specific neck (NP), thoracic pain (TP) and low back pain (LBP) represent a major clinical, social and economic burden in industrialised countries [16,22-28]. In routine practice, many treatments are used for these conditions, although very few are supported by solid evidence on efficacy, effectiveness, safety and cost/effectiveness [29-31].

Neuro-reflexotherapy (NRT) is one of the few LBP treatments supported by high quality evidence [32-35]. It involves temporary (up to 90 days) subcutaneous implantation of surgical material on specific trigger points [32-35]. The intervention is unrelated to acupuncture and is designed to deactivate the neurons involved in the mechanisms which prolong pain, neurogenic inflammation, and muscle dysfunction and contracture [20-23]. A more detailed description of the procedure and the mechanisms explaining its effect, have been published elsewhere [33]. The evidence available, including the corresponding Cochrane review, shows that NRT is safe, more effective than a sham procedure (i.e., implanting the same number of surgical devices ≤ 5 mm around the specific trigger points), and that its use in routine practice significantly increases the effectiveness and cost/effectiveness of treatment for subacute and chronic LBP in Spain, and specifically within the Spanish National Health Service [32-35]. All the evidence-based clinical guidelines which have reviewed the quality of the evidence on NRT, have rated it as “high” [29,30,36-39] In fact, NRT is the only non-pharmacological therapy for LBP for which the net benefit is considered to be “substantial” by the evidence review supporting the US evidence-based clinical guidelines for LBP [39]. Moreover, all the guidelines covering the geographical settings where the procedure was available have recommended its use in clinical practice [36-38].

The Spanish National Health Service (SNHS) provides free health care to every resident in Spain. The SNHS is managed at the regional level and fully financed by taxes, although regional governments can implement local taxes or copayments to provide additional funding. Decisions on coverage of non-pharmacological technologies can be made at the regional or national level.

In 2004, NRT was implemented in routine practice of the SNHS in the Balearic Islands (Ib-Salut) for treating subacute and chronic patients with non-specific NP, TP and LBP, in the same applications conditions and under the same post-adoption surveillance methods in which it had been previously assessed and test-piloted [32-35,40-43]. The latter were designed in such a way that they do not interfere with routine practice; clinicians routinely gather all relevant data using validated instruments (e.g., pain severity, disability, etc.), and introduce them in a software package specifically designed for that purpose [40-43].

The implementation of NRT was based on a fee-for-service; the Ib-Salut paid providers a fixed amount for each patient referred for NRT who underwent the procedure, and a different amount for each patient who was referred but did not receive NRT (e.g., when patients did not comply with indication criteria or refused to sign the informed consent). However, in 2010, the Ib-Salut and providers agreed to enhance post-adoption surveillance methods by using risk sharing tools in order to improve quality of care and to support value-based reimbursement decisions, starting January 1st, 2011. The objective of this study was to describe these tools and their results one year after their implementation.

## Methods

### Study population

**Figure 1:**
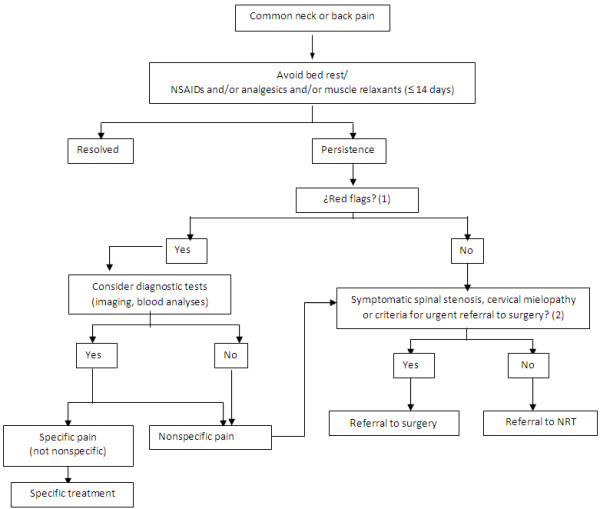
**Referral protocol for NRT intervention [**[34]**,**[40]**,**[41]**].**

In accordance with the referral protocol used in previous studies (Figure 1) [34,40,41], primary care physicians in the Ib-Salut referred patients with NP, TP and LBP in whom pain severity was ≥ 3 points on a 10-point visual analogue scale (VAS) [44], and lasted ≥ 14 days despite medication and other treatments, to certified NRT Units. These indication criteria include patients with failed back surgery. Exclusion criteria were suffering neurogenic claudication caused by lumbar spinal stenosis, cervical mielopathy or showing criteria for urgent referral to surgery, such as signs suggesting cauda equina syndrome (e.g., progressive motor weakness in the legs, sphincter disturbance, saddle anesthesia, or sensory level) [22,34,40,41].

Patients with “red flags” suggesting that pain might be caused by fractures or systematic diseases (such as fever or unexplained weight loss), could be referred to NRT only once these conditions had been discarded (Figure 1) [22,29,30,34,40,41].

All patients undergoing NRT following referral from Ib-Salut during 2011, and discharged before December 31st, 2011, were included in this study. Patients in whom adverse events required early extraction of the surgical material, were identified and excluded from the analyses.

### Shared risk tools

Following the clauses established in the contract, the provider invoiced the Ib-Salut 48.57 € for each patient referred for NRT when the procedure was not performed, and 615.28 € for each NRT intervention. These prices match those which the Ib-Salut is invoiced for in routine practice. They were based on the costs associated with NRT in the RCT which showed this technology to be cost/effective [33], and have been adjusted for inflation thereafter.

Fixed payments represented 50% of the amount invoiced. Variable payments corresponded to the remaining 50%, payable only if results reached the standards defined in the contract.

The variable amount broke down as follows: reaching the standards for clinical evolution implied payment of 50% of the variable amount; reaching the standards on the reduction of use of drugs, 30%, and reaching the standards on rates of spinal surgery, 20%. For instance, if results did not meet any of the standards, only 50% of the invoice (the fixed amount) would be paid; if only the standards on pain and disability were met, only 75% of the invoice (the fixed amount plus 50% of the variable amount) would be paid.

The Ib-Salut defined three sets of standards; clinical standards for patients’ clinical evolution; standards for the reduction of medication intake; and standards for the proportion of patients in whom spinal surgery was prescribed after undergoing NRT.

Clinical standards focused on the most important clinical outcomes for patients with NP, TP or LBP: local pain (NP, TP or LBP), referred pain, and disability [45]. The standards for each of these variables was established by the person designated by the Ib-Salut for this task (CC-A), in collaboration with the first author of the studies which had identified the cut-off point for clinical relevance for these variables in Spanish LBP and NP patients (FMK). This cut-off point corresponds to 30% of baseline score of the corresponding variable (local pain, referred pain or disability), and matches the cut-off established in other settings [46-49]. Therefore, these standards were considered to be met if the median improvement at discharge for each one of these variables was ≥ 30% of baseline score [46-49].

Standards for reduction in medication and for the proportion of spinal surgery among NRT patients, were established in three phases. Firstly, the Ib-Salut defined the ideal standards which, if met, would generate savings above the cost of NRT. Subsequently, the person designated by the Ib-Salut for developing this SRC (CC-A), In collaboration with the first author of the RCTs on NRT (FMK), verified that these standards were realistic, based on the effect size reported for NRT in: these RCTs, previous studies conducted in routine practice, the Cochrane systematic review, and the evidence-based clinical guidelines [29,32-35,39-43,50]. Finally, since the reported effect size was equal or above the ideal standards defined by the Ib-Salut, they were accepted by the provider and incorporated into the contract. Previous studies had shown that adverse events from NRT were minor (essentially, transient skin itching or limited skin infection) [32-35,40-43]. Therefore, the contract did not include standards for adverse events. Nevertheless, the number of patients in whom adverse events required the extraction of the surgical material, was documented.

Standards for the use of medication focused on patients’ intake of non-steroidal anti-inflammatory drugs (NSAIDs), steroids, non-opioid analgesic, codeine, other opioids, anti-epileptic drugs, and muscle relaxants. Only use for NP, TP or LB was considered. The Ib-Salut established these standards as either: a) for each type of drug, the number of patients using it at discharge was ≤ 80% of those using it at baseline, or b) the number of patients using any of these drugs at discharge was ≤ 80% of those who did so at baseline.

Decompressive spinal surgery has proven effective for patients in whom conservative treatment fails to significantly improve radicular pain caused by disk herniation within 6 weeks, or caused by spinal stenosis within 3–6 months [45,51-56]. Although the effectiveness of spinal fusion for LBP patients without radicular pain remains controversial [30], it is still considered when conservative treatments fail for ≥ 2 years [29]. These types of patients, are often referred to NRT as the “last resource” to avoid surgery [41,43,57]. Therefore, the Ib-Salut established that standards for the proportion of spinal surgery among NRT patients would be met if surgery, for the reason for which patients had been referred for NRT, was prescribed to ≤ 20% of the subjects who underwent the procedure.

Standards were assessed when patients were discharged, 3 months after having undergone the NRT treatment. Reaching the standards for each of the clinical outcomes (local pain, referred pain, NP-related disability and LBP-related disability) implied payment of 12.5% of the variable amount. Disability was not taken into account among TP patients because validated instruments to measure TP-related disability do not exist. Reaching the standards for reduction of medication intake implied payment of 30% of the variable amount, and reaching the standards for the proportion of NRT patients in whom spinal surgery was subsequently prescribed implied payment of 20% of the variable amount.

### Intervention

Following the application conditions in which NRT had proven effective, safe, and cost-effective [40,51,57], specialists at the NRT units assessed indication criteria. Patients complying with indication criteria were presented written informed consents for undergoing NRT and for allowing the use of their data for this study. Units in which the interventions were performed, and physicians who performed them, had been certified following specialised professional standards [58]. According to the Spanish law, this study did not need to be submitted to an Institutional Review Board.

In the event of experiencing adverse events (e.g., itching or skin infection), patients were instructed to contact the specialised unit or their primary care centre [32,34,35,40,41]. Cases in which adverse events led to early extraction of the material, were identified. In the remaining patients, twelve weeks after the intervention was performed the surgical material was extracted and indication criteria for repeating the NRT intervention were assessed. These criteria are: [34,41] having improved ≥ 2 VAS points after the first intervention, pain severity still ≥ 3 VAS points, and having patient’s written consent for repeating it. Patients not meeting these criteria were discharged.

### Outcome assessment

The outcome assessment was based on previously validated post-adoption surveillance mechanisms which are used in routine clinical practice [40,41]. Primary care physicians assessed patients’ clinical condition. Referral to the certified NRT Unit was determined by them using a standardised protocol (Figure 1) [40,41].

On patient’s first visit the following data were gathered: gender, age (date of birth); reason for referral (NP, TP or LBP); time elapsed since first diagnosis (days); duration of the current pain episode (days); and date of referral for NRT.

On the first and all subsequent visits to the primary care centre and the certified NRT Unit, the following data were gathered: severity of local pain (LP) and referred pain (RP), disability, employment status (working, receiving financial compensation for LBP, TP or NP, or passive –student, housewife, unemployed, etc.-); pregnancy; comorbidities; involvement in work-related claims; involvement in litigation; diagnostic tests undergone (X-Rays, MRI, electromyogram, etc.); test results (e.g., imaging findings), and therapeutic procedures prescribed (including drug treatment and spinal surgery).

Data on pain and disability were provided by patients. They rated these variables on their own and without assistance or interference from clinicians or auxiliary personnel. Separate 10-cm VAS were used for local pain (NP, TP or LBP) and referred pain (RP) [44]. The Spanish version of the Roland-Morris Questionnaire (RMQ) was used to score LBP-related disability [59]. The Neck Disability Index (NDI) was used to assess NP-related disability [60]. From best to worst, value ranges for these instruments are 0–10 for VAS, 0–24 for RMQ and 0–100 for NDI [44,48,59].

Data were introduced into a database by auxiliary staff independent from the clinicians. Clinicians had access to these data (since they are useful for clinical decisions –e.g., repeating the NRT intervention-), but could not alter them.

Every month, patients who were discharged were identified, and data on their clinical status, drug use and prescription of surgery during the follow-up period were transferred to the team in charge of analysing results.

### Analysis

Analysis of results was performed by a team who had no contact with the clinicians involved in performing the interventions or with the administrative staff in charge of payments at the Ib-Salut.

Absolute and relative frequencies were calculated for categorical variables. Values for continuous variables were described through their median and interquartile range (IQR).

According to the available evidence, data on duration of the current pain episode were categorised as subacute (14–90 days), and chronic pain (> 90 days) [61,62]. There is no evidence for categorising the time elapsed since first diagnosis. Data on this variable were categorised as ≤ 1 year, 1–5 years, 5–10 years, and > 10 years.

Analyses were limited to data from patients who had undergone NRT, excluding those who had been referred for the procedure but did not undergo it (e.g., those not complying with indication criteria), and those in whom adverse events required early extraction of the surgical material implanted. The total number of these patients was registered.

At the design phase of this study, it was decided that the number of patients with missing values for data relevant to assess whether the standards had been met (e.g., patients not answering the VAS, RMQ or NDI questionnaires) would be registered and, if ≥ 10%, a sensitivity analysis would be performed assuming that all missing data had been failures.

In order to assess whether standards were met, the following procedures were used. The median and IQR of VAS scores for local pain (NP, TP or LBP) at baseline and at the end of the follow-up period were calculated. The same procedure was repeated for the VAS scores for referred pain, the RMQ scores for LBP-related disability (including only data from patients referred to NRT for LBP) and NDI scores for NP-related disability (including only data from patients referred to NRT for NP). The number of patients who were using NSAID, steroids, non-opioid analgesic, codeine, other opioids, anti-epileptic drugs, and muscle relaxants when referred for NRT and at discharge, was determined. The number of patients using any kind of drug at baseline and discharge, was also identified. Finally, patients in whom spinal surgery had been prescribed for the reason why they had previously been referred to NRT, were identified.

The number of patients with missing data (e.g., patients who did not answer the instruments to assess pain and disability at baseline and at discharge) was also identified.

Since outcome assessment occurred 3 months after performing the NRT intervention, variable payments related were delayed for at least that period. Therefore, in order to prevent delays in payments which might have compromised feasibility of the contract, invoicing and preliminary assessment of results were performed monthly from April onwards, based on the data from patients discharged during the previous month. Analyses comprising all data of patients treated during 2011 were repeated in April 2012, when data and outcomes for all NRT interventions performed during that year became available.

Each month, the analysts assessed whether the standards had been met and listed the names of the patients discharged, grouping those who complied and did not comply with each of the standards, separately. The administrative staff of the Ib-Salut had full access to the database containing all patients’ data, checked that data on patients’ use of medication and prescription of surgery coincided with those from the Ib-Salut’s database, double-checked that lists of patients complying and not complying with the standards was correct, and verified that the global assessment of whether each standard was met, was correct. It had been planned that if discrepancies or doubts arose, providers, analysts and the administrative staff of the Ib-Salut would meet to discuss them. All data were transferred to the person designated by the Ib-Salut to monitor results from NRT (CC), who authorised the administrative of the Ib-Salut to pay the amount corresponding to the standards which had been met.

According to the post-adoption surveillance mechanisms, data were routinely captured in daily practice [40,41]. Therefore, analyses only involved assessing whether results matched the standards established in the contract, which required less than 30 minutes per month. The team responsible for data analysts was employed full-time and paid a fixed monthly salary. Hence, the cost of conducting the analyses was considered negligible and was not measured.

## Results

During 2011, 909 patients referred by the Ib-Salut for NP (244 patients), TP (61) and LBP (604), underwent NRT and were discharged before the 31st of December 2011. All patients complying with indication criteria for NRT, signed their informed consents.

Adverse events required early extraction of the surgical material in only 38 (4.2%) patients. All adverse events consisted of skin reactions; in 7 patients, they were triggered by a previously unknown allergy to metal, and in 31 cases they consisted of recurrent or persistent local skin infection, despite topical treatment with an antibiotic cream. None of the cases required systemic treatment and the reaction disappeared after the surgical devices were extracted in all cases.

Therefore, 871 patients were included in this study. Their characteristics are shown in Table 1.

**Table 1:** Patient characteristics (n = 871)

**Variables**	**N**	**Value**
Gender (male) *	871	270 (31.0
Age (years) ^¥^	871	56 (45; 67
Reason for referral to NRT *	871	
Neck Pain (NP)		235 (26.9)
Thoracic pain (TP)		60 (6.9)
Low Back Pain (LBP)		576 (66.2)
Employment status *		
Passive (students, housewife, etc.)	535	320 (59.8)
Receiving financial compensation for LBP, TP or NP		34 (6.3)
Working		215 (40.2)
Duration of pain, since first diagnosis (days) ^¥^	763	2,555 (730; 4,380)
Duration of pain since first diagnosis, categorised *	763	
≤ 1 year		136 (17.8)
1-5 years		219 (28.7)
5-10 years		204 (26.7)
>10 years		204 (26.7)
Duration of current pain episode (days) ^¥^	809	180 (60; 365)
Duration of current pain episode (days), categorised *	809	
Subacute (≤ 90 days)		309 (38.2)
Chronic (>90 days)		500 (61.7)
Pregnancy *		2 (0.3)
Other comorbidities *	601	677 (84.4)
Involved in work-related claims *	802	14 (3.3)
Involved in litigation *	427	8 (1.9)
Baseline severity of local pain (NP, TP or LBP) (VAS score) ^¥^	426	7.0 (6.0: 9.0)
	870	660 (75.8)
Reporting referred pain at baseline*	871	6.0 (2.0; 8.0)
Baseline severity of referred pain (VAS score) ^¥♦^	660	13 (9.0; 17.0)
Baseline lumbar disability (RMQ score) ^¥♦^	574	40 (30.0; 50.0)
Baseline neck disability (NDI score) ^¥♦^	229	
Diagnostic procedures undergone during the current pain episode, before being referred for NRT*		69 (7.9)
X-Ray	871	98 (11.2)
MRI	871	21 (2.4)
Other¤	871	
Imaging findings *		698 (80.1)
Disc degeneration	871	137 (15.7)
Facet joint degeneration	871	68 (7.8)
Scoliosis	871	3 (0.3)
Difference in leg length	871	14 (1.6)
Spondylolisis	871	50 (5.7)
Spondylolisthesis	871	87 (9.9)
Spinal stenosis	871	229 (26.3)
Disc protrusion	871	340 (39.0)
Disc herniation (extrusion)	871	419 (48.1)
Other findings ^∞^	871	30 (3.4)
No findings	871	
Ongoing treatments when referred for NRT		751 (86.2)
Drugs*	871	721 (82.8)
Analgesics		712 (81.7)
Non-opioid		0 (0.0)
Codeine		78 (9.0)
Other opioids		568 (65.2)
NSAIDs		25 (2.8)
Steroids		132 (15.1)
Muscle relaxants		164 (18.8)
Other^ɣ^		
Non pharmacological treatments*	871	42 (4.9)
Physical therapy/Rehabilitation		7 (0.8)
Had undergone surgery for the current episode, before being referred for NRT*		

NP: Neck Pain TP: Thoracic pain LBP: Low back pain.

VAS: Visual Analogue Scale (range from better to worse; 0–10).

RMQ: Roland-Morris Questionnaire (range from better to worse: 0–24).

NDI: Neck Disability Index (range from better to worse: 0–100).

NRT: Neuro-reflexotherapy intervention.

*Frequency (%); ¥ Median (P25; P75);

¤Other diagnostic procedures: EMG, CT scan, scintigraphy.

∞Other imaging findings: annular tear, loss of cervical lordosis, loss of thoracic cifosis, loss of lumbar lordosis, horizontalization of the sacrum, lumbarization of S1, sacralization of L5.

^♦^ In patients in whom the variables “referred pain”, “RMQ” and “NDI” were applicable (660 patients who reported having referred pain, 235 who were referred for neck pain and 576 who were referred for low back pain, respectively).

^ɣ^ Other drugs: anti-epileptics or antidepressants prescribed for treating pain.

Some patients did not respond to the questionnaires on baseline severity of local pain (one patient), LBP-related disability (two patients) and NP-related disability (six patients). At discharge, some patients left some questionnaires unanswered; the VAS for local pain (one patient), the VAS for referred pain (three patients), the questionnaire assessing LBP-related disability (RMQ) (three patients), and the one assessing NP-related disability (NDI) (16 patients). Some patients left some questionnaires unanswered at baseline and discharge. The numbers for VAS for local pain, VAS for referred pain, RMQ and NDI were one, two, two and five, respectively. In total, twenty-four patients (2.8%) failed to respond to at least one of the questionnaires. Data on medication were checked against invoicing data held at the Ib-Salut, and data on prescription of surgery were checked against hospital registries. Therefore, there were no missing data for these variables.

Standards established in the contract on patients’ clinical evolution, use of drugs and surgery, were met, both at the yearly analysis (Tables 2, 3 and 4) and the monthly analyses (data not shown). In the yearly analysis, the difference between median severity of local pain at baseline and at discharge was 57.1%. Corresponding values for referred pain, LBP-related disability and NP-related disability were 75.0%, 53.8.0% and 45.0% (Tables 2, 3 and 4). Patients taking medication for the reason they were referred to NRT, were 751 upon referral, and 218 (29.0%) at discharge. Spinal surgery was not prescribed to any of the patients who underwent NRT during 2011 (Tables 2, 3 and 4).

**Table 2:** Results on clinical outcomes (N=871)

**Clinical outcome**^¥^	**N**^¤^	**Baseline score**	**Score at discharge**	**Improvement**
Local pain (NP, TP or LBP) (VAS)	870	7 (6–9)	3 (1–5)	57.1%
Referred pain (VAS) ^♦^	657	6 (2–8)	1.5 (0–4)	75.0%
LBP-related disablity (RMQ) ^♦^	573	13 (9–17)	6 (2–12)	53.8%
NP-related disability (NDI) ^♦^	219	40 (30–50)	22 (15–34)	45.0%

NP: Neck pain; TP: Thoracic pain; LBP: Low back pain.

¥ Median (P25; P75).

¤ Number of patients who answered the questionnaires at baseline and when discharged.

^♦^ In patients in whom the variables “referred pain”, “RMQ” and “NDI” were applicable (660 patients who reported having referred pain, 576 who were referred for low back pain, and 235 who were referred for neck pain, respectively).

VAS: Visual Analogue Scale (range from better to worse: 0–10).

RMQ: Roland-Morris Questionnaire (range from better to worse: 0–24).

NDI: Neck Disability Index (range from better to worse: 0–100).

**Table 3:** Results on the use of medication (N=871)

**Patients using medication**	**At baseline**	**At discharge**	**Proportion of patients using medication at discharge**
Any type	751	218	29.0%
NSAIDs	568	125	22.0%
Analgesics*	712	198	27.8%
Muscle relaxants	132	13	9.9%
Other drugs^ɣ^	145	32	11.9%

* Includes opioids and non-opioids.

^ɣ^ Other drugs: anti-epileptics or antidepressants prescribed for treating pain.

**Table 4:** Results on rates of spinal surgery prescribed after NRT intervention

**Patients who underwent NRT**	**Patients in whom surgery was prescribed after having undergone NRT**	%
871	0	0

There were no discrepancies between providers, analysts and the administrative staff of the Ib-Salut. Given these results, the Ib-Salut paid 100% of the amount invoiced for NRT interventions.

## Discussion

Shared risks contracts (SRCs) have taken center stage in the regulatory framework related to the adoption of new technologies, and numerous publications have described them in depth, reporting many obstacles for their generalisation into routine practice [63-70]. These obstacles include: providers and the industry can perceive SRCs as a threat to their income, which may stifle research and innovation; payers may be unenthusiastic because of the added burden in their administrative workload; in the case of certain technologies, concerns may exist over the balance between potential savings and monitoring costs; follow-up of relevant outcomes through validated instruments in routine practice may be difficult; and SRCs may be used for implementing technologies which lack evidence on effectiveness in specific geographic areas, while patients are denied access to them in other settings, leading to inequities across health areas within a single Health Service [13-18,71,72].

NRT was selected for exploring the feasibility and effectiveness of using risk sharing tools for quality control and value-based reimbursement of a non-pharmacological technology in Spain, because it complied with the following three requisites: a) the efficacy, effectiveness and cost/effectiveness of the procedure had been assessed through high quality RCTs and studies in clinical practice [32-35,40-43], which made it feasible for the contract to establish evidence-based, realistic standards, b) it had been implemented in routine practice in the same application conditions which were used in those studies, with the same indication criteria and education and training standards [32-35,40-43,58], c) post-adoption surveillance mechanisms already existed and had shown to be comprehensive and valid [40-42], which made it possible to use risk sharing tools at virtually no additional cost. Generalising this approach to technologies which do not comply with these pre-requisites may be difficult and require additional costs. However, results from the current study show that it is indeed feasible to use this approach for a non-pharmacological health technology, and set a precedent for exploring the feasibility of generalising this approach to other treatments for NP, TP and LBP, and potentially treatments for other conditions across the SNHS.

Many treatments for NP, TP and LBP, such as some rehabilitation or surgical procedures [1,30], are not supported by solid evidence on effectiveness and cost/effectiveness, but are nevertheless used in routine clinical practice, both in Europe and the US, without any post-adoption surveillance mechanisms. Applying the approach described in this study to these technologies may prove challenging, but may also lead to larger savings and potentially better outcomes and reduced risks for patients. In fact, available data suggest that, for instance, the use and cost of spinal fusion for common low back pain are soaring, without any evidence that this increase is associated with an improvement in clinical outcome [26,73]. Implementing post-marketing surveillance mechanisms for each of these technologies separately may be more efficient in the case of the most expensive technologies, such as spinal fusion [26], than in the case of low-cost ones, such as some physical therapy treatments [1]. A more efficient strategy might be to establish a prospective registry for patients with NP, TP and LBP, gathering data on all procedures relative to their condition. In fact, one of the top priorities recommended by the Institute of Medicine for investing the $1.1 billion devoted to comparative effectiveness research by the 2009 American Recovery and Reinvestment Act, was to “establish a prospective registry to compare the effectiveness of treatment strategies for low back pain without neurological deficit or spinal deformity” [74]. A pilot test in 17 primary care and specialty centres, shows that it is feasible to implement a valid registry for these patients within the SNHS [75]. Generalising this registry might facilitate the use of risk sharing tools for quality control and value-based reimbursement.

The follow-up period in this study was 3 months, which may be seen as relatively short. However, NRT focuses on treating the current pain episode, not on preventing relapses, and this period appears to be clinically relevant and appropriate for this purpose. In fact, the prognosis of chronic low back pain is determined by changes in pain and disability occurring in the initial period [76]. Moreover, previous studies have shown that NRT can be repeated if relapses occur, although the proportion of subjects who suffer from relapse after NRT within three years, is below 10% [41]. In addition, three months is the clinical follow-up period which has been used in most studies on NRT in routine practice and, therefore, the follow-up period for which data on results to be expected were available when this study was developed [40-43]. Finally, studies with NP and LBP patients conducted in routine practice within the Spanish National Health Service have shown that losses to follow-up are minimal up to 3 months [34,43,60,61,77,78], but start to rise at 6 months [61,77-79], and are substantial thereafter [34,78]. For these reasons, a 3-month follow-up period was deemed appropriate when designing this study.

Validity of data determining whether standards established in the agreement have been met (Tables 2, 3 and 4) should be discussed. Patients’ clinical evolution was assessed through previously validated methods [44,48,59], and are consistent with data from previous RCTs and studies in clinical practice [32-35,40-43]. Data on use of medication and rates of spinal surgery was shown to be accurate after having been checked against the Ib-salut activity and pharmacy registries. The later include data on all drugs prescribed as well as those dispensed and invoiced by pharmacies. Moreover, standards on use of medication and surgery rates which were agreed upon, were calculated to ensure that funds invested by the Ib-Salut in NRT were cost-effective.

High rates of excluded patients, losses to follow-up and missing data, may introduce bias; in this study, less than 5% of the patients who underwent NRT were excluded because adverse events required early extraction of the surgical material, there were no losses to follow-up, and missing data were below 3% (Tables 1, 2, 3 and 4). These features suggest that generalisability of these results to LBP patients treated with NRT within the SNHS should not be a concern.

The provider of NRT for the Ib-Salut is a not for profit institution which specialises in NP, TP and LBP research and, in fact, is responsible for most Spanish research in this field [57]. The staff of the Ib-Salut in charge of post-adoption surveillance for NRT, members of the team analysing results, researchers involved in this study, and clinicians performing NRT are paid a fixed salary and have no economic incentives for applying the procedure in cases in which it is not indicated, or for biasing analysis of results. The use of post-adoption surveillance and shared risk tools in routine practice may be more difficult when for-profit providers are involved, in settings where these conditions are not met, or in which clinicians are paid on a fee-for-service basis [13-15]. Nevertheless, the potential of savings may be larger in these settings.

Randomised controlled trials (RCTs) are the gold standard for assessing the efficacy, effectiveness and cost/effectiveness of any health technology. In an ideal world, all health technologies would be assessed through high quality RCTs before being used in the clinical environment, and post-adoption surveillance methods would be applied from the start of their use in routine practice. However, strong incentives against this approach exist and, as many examples show, many technologies are implemented in routine practice in the absence of such an evidence, or even disregarding the evidence against implementation [2,3,80]. No form of post-adoption surveillance can substitute the evidence deriving from RCTs, but using post adoption surveillance combined with risk sharking tools for all technologies that lack long term effectiveness and safety data and for which costs of usage are higher than monitoring costs, is likely to improve the efficiency of health resources.

This approach is especially needed for technologies which lack the support of robust evidence on efficacy, effectiveness. In the field of NP and LBP this includes, for instance, ultrasound for LBP, most forms of conservative treatment for lumbar spinal stenosis or spinal fusion for common LBP [26,30,53,73,81,82]. Moreover, even in the case of technologies for which there is evidence on effectiveness or cost/effectiveness, this approach would pave the way to comparative effectiveness research and would likely contribute to reducing inappropriate use (including off-label use, inappropriate referral, over-prescription of drugs and slim clinical outcomes in some subgroups of patients), since it would financially penalize any worsening in outcomes. Examples within the same field in which this approach would be suitable, include the inappropriate use of magnetic resonance imaging, or the prescription of spinal surgery in cases other than symptomatic disc herniation or spinal stenosis complying with surgical criteria [51-53,55,56,83,84].

The absence of guidelines regarding the use of SRC and the expected increase in its use in the near future in Spain, calls for regulatory action. Some preliminary work has been done in this direction [85].

## Conclusions

Results from this experience show that it is feasible an effective to combine post adoption surveillance methods with risk sharing tools for controlling quality of care and support value-based reimbursement decisions for NRT. The feasibility of generalizing this approach to other settings and to other non-pharmacological treatments should be explored, since this is likely to improve the efficiency of health resources.

## Competing interests

The authors declare that they have no competing interests, and that no funds were received for conducting this study.

## Authors’ contributions

The authors have contributed equally to the design, analysis, interpretation and conclusions of this study. Both authors read and approved the final manuscript.

## Pre-publication history

The pre-publication history for this paper can be accessed here:

http://www.biomedcentral.com/1472-6963/13/181/prepub
